# Does Confinement Affect Treatment Dropout Rates in Patients With Gambling Disorder? A Nine-Month Observational Study

**DOI:** 10.3389/fpsyg.2021.761802

**Published:** 2021-12-14

**Authors:** Isabel Baenas, Mikel Etxandi, Ester Codina, Roser Granero, Fernando Fernández-Aranda, Mónica Gómez-Peña, Laura Moragas, Sandra Rivas, Marc N. Potenza, Anders Håkansson, Amparo del Pino-Gutiérrez, Bernat Mora-Maltas, Eduardo Valenciano-Mendoza, José M. Menchón, Susana Jiménez-Murcia

**Affiliations:** ^1^Department of Psychiatry, Bellvitge University Hospital-IDIBELL, Barcelona, Spain; ^2^Ciber Fisiopatología Obesidad y Nutrición (CIBERObn), Instituto de Salud Carlos III, Madrid, Spain; ^3^Department of Psychobiology and Methodology, Autonomous University of Barcelona, Barcelona, Spain; ^4^Department of Clinical Sciences, School of Medicine, University of Barcelona, Barcelona, Spain; ^5^Psychiatry and Mental Health Group, Neurosciences Programme, Instituto de Investigación Biomédica de Bellvitge (IDIBELL), Barcelona, Spain; ^6^Department of Psychiatry and Child Study Center, Yale University School of Medicine, New Haven, CT, United States; ^7^Connecticut Council on Problem Gambling, Wethersfield, CT, United States; ^8^Connecticut Mental Health Center, New Haven, CT, United States; ^9^Department of Neuroscience, Yale University, New Haven, CT, United States; ^10^Department of Clinical Sciences Lund, Psychiatry, Faculty of Medicine, Lund University, Lund, Sweden; ^11^Region Skåne, Gambling Disorder Unit, Malmö, Sweden; ^12^Department of Public Health, Mental Health and Perinatal Nursing, School of Nursing, University of Barcelona, Barcelona, Spain; ^13^Ciber Salut Mental (CIBERSAM), Instituto de Salud Carlos III, Madrid, Spain

**Keywords:** COVID-19, confinement, gambling disorder, dropout, coping

## Abstract

**Background and Aims:** COVID-19 pandemic and confinement have represented a challenge for patients with gambling disorder (GD). Regarding treatment outcome, dropout may have been influenced by these adverse circumstances. The aims of this study were: (a) to analyze treatment dropout rates in patients with GD throughout two periods: during and after the lockdown and (b) to assess clinical features that could represent vulnerability factors for treatment dropout.

**Methods:** The sample consisted of *n*=86 adults, mostly men (*n*=79, 91.9%) and with a mean age of 45years old (*SD*=16.85). Patients were diagnosed with GD according to DSM-5 criteria and were undergoing therapy at a Behavioral Addiction Unit when confinement started. Clinical data were collected through a semi-structured interview and protocolized psychometric assessment. A brief telephone survey related to COVID-19 concerns was also administered at the beginning of the lockdown. Dropout data were evaluated at two moments throughout a nine-month observational period (T1: during the lockdown, and T2: after the lockdown).

**Results:** The risk of dropout during the complete observational period was *R*=32/86=0.372 (37.2%), the Incidence Density Rate (*IDR*) ratio T2/T1 being equal to 0.052/0.033=1.60 (*p*=0.252). Shorter treatment duration (*p*=0.007), lower anxiety (*p*=0.025), depressive symptoms (*p*=0.045) and lower use of adaptive coping strategies (*p*=0.046) characterized patients who abandoned treatment during the lockdown. Briefer duration of treatment (*p*=0.001) and higher employment concerns (*p*=0.044) were highlighted in the individuals who dropped out after the lockdown. Treatment duration was a predictor of dropout in both periods (*p*=0.005 and *p*<0.001, respectively).

**Conclusion:** The present results suggest an impact of the COVID-19 pandemic on treatment dropout among patients with GD during and after the lockdown, being treatment duration a predictor of dropout. Assessing vulnerability features in GD may help clinicians identify high-risk individuals and enhance prevention and treatment approaches in future similar situations.

## Introduction

The COVID-19 pandemic, declared as a public health emergency by the World Health Organization [World Health Organization (WHO), 2020], has affected more than 200 countries around the world since the first case appeared in Wuhan (China) in December 2019 (Yuki et al., 2020). Spain has been considered one of the most affected countries by this pandemic since the early stages. Its first case of infection was reported within the first months of 2021, and since then, the number of cases has continued to increase. Along with other European countries, such as Italy and France, Spain has presented one of the highest mortality rates due to the pandemic (Ceylan, 2020).

To control pandemic spread, confinement and other restrictive measures have been stated everywhere (Hale et al., 2020), being social distancing, disruption of daily routines, and economic and employment uncertainty reported as some of the most frequent COVID-19-related concerns (Chew et al., 2020). This context alongside with health worries due to the pandemic has led to an increase in anxiety and depressive symptoms among general population (Islam et al., 2020; Wang et al., 2020). In this vein, people with a mental illness, such as gambling disorder (GD), have been considered at particularly high risk of suffering the psychological impact of the COVID-19 pandemic (González-Sanguino et al., 2020; Özdin and Bayrak Özdin, 2020; Rajkumar, 2020).

GD is defined as engaging in a repeated compulsive gambling behavior, accompanied by unsuccessful efforts to stop the behavior and uncontrollable urges to keep gambling, which leads to considerable distress and impairment. This gambling behavior tends to persist over time despite negative consequences on the person’s physical, mental, social, or financial well-being [American Psychiatry Association (APA), 2013]. GD was included within the “substance-related and addictive disorders” category in the fifth edition of the Diagnostic and Statistical Manual of Mental Disorders [DSM-5; American Psychiatry Association (APA), 2013]. It has been considered the most prevalent behavioral addiction, with a lifetime prevalence ranging from 1.1 to 10.6% (Calado and Griffiths, 2016). In Europe, epidemiological studies have shown that gambling problems range from 0.3 to 3.3% (Dirección General de Ordenación del Juego (DGOJ) 2015).

In the face of environmental changes, maladaptive coping strategies and behaviors, such as those related to gambling activity, could be used to cope with psychological distress (Asmundson and Taylor, 2020; Rajkumar, 2020; Fernández-Aranda et al., 2020a; Avena et al., 2021), specifically in individuals with higher vulnerability (e.g., those with a mental illness; Fernández-Aranda et al., 2020a; Baenas et al., 2020).

During confinement, the use of technologies has extended to more aspects of daily life. Hence, excessive engagement in specific online activities, such as gambling, may lead to severe problems and increases the risk of a disordered or addictive use (King et al., 2020; Mestre-Bach et al., 2020). These maladaptive behaviors are usually presented in combination with social isolation and boredom, factors which have been also linked to confinement and previously associated with the pathogenesis of GD (Ledgerwood and Petry, 2006; Mercer and Eastwood, 2010). On the other hand, the use of gambling has been also stated as a mechanism to deal with adverse economic circumstances in prior financial crises (Olason et al., 2015; Economou et al., 2019). Similarly, economic difficulties due to pandemic and confinement might represent a risk factor that could favor gambling activity. Moreover, some works have described a risk profile for engaging in gambling behavior during confinement (being young male regular gambler with high alcohol use prior to confinement; Håkansson, 2020a; Emond et al., 2021; Hodgins and Stevens, 2021). In the non-clinical study by Price (2020), online gambling modality was associated with the subgroup of gamblers at a higher risk of developing GD, characterized by greater psychological impact due to pandemic and substance use while gambling. In this line, gambling behavior prior to the pandemic predicted gambling activity during confinement, mainly at the expense of online gambling (Gainsbury et al., 2020; Håkansson, 2020b; Emond et al., 2021; Hodgins and Stevens, 2021).

With reference to the potential of the pandemic worsening gambling problems (King et al., 2020; Marsden et al., 2020), legal measures have been adopted by governments in an attempt to minimize these consequences. For instance, in Spain, advertising related to gambling has been limited (SBC News, 2020). Among other useful tools, a consensus guidance on how to avoid problematic use of the internet during the pandemic has been also published (Király et al., 2020). In this guidance, healthy general practices, such as those related to exercise or sleep hygiene were recommended, as well as specific tips regarding Internet use. Monitoring and regulating screen time exposure in children and adults, using digital well-being apps and analogue technical tools, keeping social contact with relatives and friends, or seeking professional help when needed were highly advised (Király et al., 2020).

Among people with GD, the challenges represented by the pandemic and confinement (Håkansson et al., 2020; King et al., 2020) were based on different vulnerability aspects (Allami et al., 2021; Hodgins and Stevens, 2021). For instance, higher levels of anxiety, depressive symptoms and hostility have been described in subjects with GD during confinement, in comparison with the general population (Salerno and Pallanti, 2021). In this context, gambling behavior could be conducted (and reinforced) to regulate emotions (Barrault et al., 2019), being a trigger for relapse in those patients who remained abstinent (Campbell, 2020). However, restrictive strategies and some policies adopted during confinement seemed to reduce gambling opportunities, specifically at offline locations, promoting abstinence (Donati et al., 2021). In this line, patients already in treatment for GD reported a positive attitude about restrictions due to pandemic and related to gambling activity, with improved social and familiar environments. This positive social reinforcement also contributed to maintain abstinence. Interestingly, large-scale migration from offline to online gambling has not been observed so far among patients with GD during confinement (Donati et al., 2021).

Works regarding GD treatment during confinement did not report an increase in treatment-seeking for GD, at least in the short term (Gunstone et al., 2020; Donati et al., 2021; Håkansson et al., 2021). When talking about GD treatment, cognitive-behavioral therapy (CBT) and group-based modalities have been widely demonstrated as effective options for the management of this disorder (Gooding and Tarrier, 2009; Cowlishaw et al., 2012; Jiménez-Murcia et al., 2015). Besides, internet-based treatments for GD demonstrated similar efficacy to traditional treatments (Hedman et al., 2012; Chakrabarti, 2015). Trying more flexible approaches to patient care in the face of pandemic situation (Marsden et al., 2020), tele-psychiatry has shown to be helpful (Hollander and Carr, 2020) and internet-based tools for GD have been used in some treatment facilities during confinement (Columb et al., 2020).

Within the treatment outcome in GD, dropout has been defined as leaving before the completion of a predetermined program (Milton et al., 2002; Robson et al., 2002). Despite a broad dropout range (14–50%) reported by previous national and international studies (Melville et al., 2007; Smith et al., 2010; Jimenez-Murcia et al., 2012), an average of around 26% has been agreed (Melville et al., 2007). Prior to confinement, a recent national study (Jiménez-Murcia et al., 2019) reported dropout rates of 32.4% among patients with GD regarding clinical trajectories based on GD severity among patients following a 12months manualized CBT program. Features such as patients’ medical or family issues, length of therapy, personality traits (e.g., perseverance, reward sensitivity, sensation-seeking), younger age, lower educational level, and neurocognitive variables, among others, have been studied as potential predictors of treatment dropout (Melville et al., 2007; Álvarez-Moya et al., 2011; Jiménez-Murcia et al., 2015; Mallorquí-Bagué et al., 2018, 2019; Mestre-Bach et al., 2019).

So far, most published studies concerning gambling activity during confinement focused on non-clinical populations (Auer et al., 2020; Brown and Hickman, 2020; Gunstone et al., 2020; Lindner et al., 2020; Zamboni et al., 2021). Scarce research on the effects of pandemic on GD treatment outcome and possible associated factors has been reported (Price, 2020; Donati et al., 2021). Furthermore, information related to initial stages of confinement provided a limited knowledge not only regarding its longer-term impact, but also in distinguishing its effects during time periods characterized by different levels of restrictive measures, such as the lockdown and the following months.

To the best of our knowledge, this is the first observational clinical study regarding the effect of the pandemic on GD treatment outcome related to dropout rates among clinical population. The longitudinal nature of the present work favors reporting data comparing two well-established periods of time across confinement (i.e., during and after the lockdown). Finally, the already existing involvement of these patients with the treatment Unit and a suitable observational period allow for evaluating clinical features possibly associated with dropout. Thus, the main aims of this study were to analyze treatment dropout rates in patients with GD in the two established periods, and assess clinical features that would be linked to treatment dropout in both periods.

## Materials and Methods

### Participants

The sample consisted of *n*=86 adults, mostly men (*n*=79, 91.9%) and with a mean age of 45years old (*SD*=16.85). Participants had been diagnosed according to DSM-5 criteria [American Psychiatry Association (APA), 2013] and were voluntarily recruited in the region of Catalonia (Spain), specifically at the Behavioral Addiction Unit within the Psychiatry Department of a Public University Hospital in Barcelona (Spain). All the patients recruited in the present study were already enrolled in the outpatient treatment program when confinement started. The mean duration of treatment at this point, considering therapy phase and follow-up was 14.3months (*SD*=9.2months) and around 10.5% (*n*=9) were not abstinent before the lockdown.

The outpatient treatment program offered in the Unit was based on a standardized protocol (Jiménez-Murcia et al., 2006). It has shown adequate effectiveness for GD in both short- and medium-terms, and has been widely described in previous Unit’s studies related to treatment of GD (Jiménez-Murcia et al., 2006, 2007, 2019; Jimenez-Murcia et al., 2012). It comprised (a) two initial baseline assessment visits, (b) CBT group therapy phase, and (c) a post-therapy follow-up. In the sessions prior to the treatment phase, a semi-structured clinical interview was conducted by expert psychologists and psychiatrists of our Unit with high experience in the field of behavioral addictions (SCID-5; First et al., 2015). Moreover, a protocolized psychometric assessment was also completed in a second session lasting about 90min (Jiménez-Murcia et al., 2006). The treatment phase was composed of 16 weekly sessions with a mean duration of 90min per session, conducted by an experienced clinical psychologist. The main treatment objective was training patients to put into practice CBT strategies to achieve full and definitive abstinence from gambling. Among other techniques, psychoeducation, stimulus control, response prevention, cognitive restructuring, reinforcement and self-reinforcement, skills training, and relapse-prevention techniques were used. Once completed, a post-treatment evaluation was performed, and a 24-month follow-up was initiated before being discharged from the Unit. The therapists recorded dropouts and relapses based on patients’ oral reports and written diaries and relatives’ collateral information. The presence of a co-therapist (e.g., partner, family member, and close friend) was highly recommended.

#### Contextual Setting Due to the Pandemic

The officially established COVID-19 lockdown period in the region of Catalonia (Spain) extended from March 14 to May 11, 2020. This period was characterized by a stay-at-home policy with measures such as the interruption of all non-essential work, academic, social and leisure activities outside. The coverage of basic products was guaranteed, as well as emergency medical assistance.

During the nine-month observational period of the present study, the described treatment schedule was maintained in each case. However, specific modifications were carried out due to the pandemic situation and restrictive measures. During the lockdown (i.e., March 14, 2020–May 11, 2020), face-to-face treatment was adapted to virtual modality using internet-based tools. Tutorial information was provided to guarantee accessibility and a proper use. Eventually, the presence of the co-therapist was also ensured. After the lockdown, usual face-to-face treatment was resumed (i.e., after May 11, 2020).

### Procedure

All the assessments apart from the *Brief Telephone Survey related to COVID-19* (Behavioral Addictions Unit, Psychiatry Department, University Hospital, Barcelona, Spain, 2020) were part of the standardized assessment within the Unit’s treatment program previously described (Jiménez-Murcia et al., 2006), and contemplated in other studies related to treatment of GD (Jiménez-Murcia et al., 2006, 2007, 2019; Jimenez-Murcia et al., 2012).

*The Brief Telephone Survey related to COVID-19* was obtained in the first therapeutic contact with each patient within the first 2months of confinement in Spain (i.e., lockdown period), mostly at the beginning of the lockdown. Over the nine-month observational period of this study, information regarding dropout (bivariant dropout/non-dropout) was collected by reviewing therapists’ clinical reports. Two dropout periods were distinguished: (T1) between March 14 and May 11 (i.e., during the lockdown), and (T2) between May 12 and December 31, 2020 (i.e., after the lockdown).

### Assessments

#### At Baseline

*South Oaks Gambling Screen* (SOGS; Lesieur and Blume, 1987): a 20-item instrument for screening for past-year gambling problems and related negative consequences. The total score obtained as the sum of the scored items has been used as a measure of problem-gambling severity, with a score of 5 or more suggestive of “probable pathological gambling.” The Spanish validation of the scale achieved very good psychometric results (test–retest reliability *R*=0.98, internal consistency *α*=0.94 and convergent validity *R*=0.92; Echeburúa et al., 1994). The internal consistency for this scale in the study sample was good (*α*=0.74).

*Diagnostic Questionnaire for Pathological Gambling According to DSM criteria* (Stinchfield, 2003): a self-report questionnaire with 19 items coded in a binary scale (yes-no), used for diagnosing GD according to the DSM-IV-TR [American Psychiatry Association (APA), 2000] and DSM-5 [American Psychiatry Association (APA), 2013] criteria. The Spanish adaptation of the questionnaire obtained satisfactory psychometric properties (Cronbach’s alpha *α*=0.81 for a population-based sample and *α*=0.77 for a clinical sample; Jiménez-Murcia et al., 2009). The internal consistency for this scale in the study sample was good (*α*=0.83).

*Symptom Checklist-90-Revised* (SCL-90-R; Derogatis, 1990): a 90-item self-report questionnaire measured on an ordinal 3-point scale. It evaluates a broad range of psychological problems and psychopathology, based on nine primary symptom dimensions (Somatization, Obsession-Compulsion, Interpersonal Sensitivity, Depression, Anxiety, Hostility, Phobic Anxiety, Paranoid Ideation, and Psychoticism). It includes three global indices (global severity index, positive symptom distress index, and total positive symptom). The validation of the scale in a Spanish population (Derogatis, 2002) obtained a mean internal consistency of *α*=0.75. The internal consistency in the study was between good (*α*=0.77 for paranoid ideation) to excellent (*α*=0.98 for global indexes).

*Temperament and Character Inventory-Revised* (TCI-R; Cloninger, 1999): a questionnaire with 240-items scored on a 5-point Likert scale and measuring personality derived from three character dimensions (Self-Directedness, Cooperativeness, and Self-Transcendence) and four temperament dimensions (Harm Avoidance, Novelty Seeking, Reward Dependence, and Persistence). Evaluation of the Spanish revised version (Gutiérrez-Zotes et al., 2004) had an internal consistency of *α*=0.87. We obtained verbal consent from the author for using the TCI-R questionnaire in a public non-profit hospital and exclusively linked to the field of research. This questionnaire was administered in its Spanish adaptation, in which the original author participated (Gutiérrez-Zotes et al., 2004). The internal consistency in the study was between good (*α*=0.72 for novelty seeking) to excellent (*α*=0.90 for persistence).

*Other variables*: Additional data (e.g., socio-demographic, and socio-economic) were measured. They were collected in a semi-structured face-to-face clinical interview as described elsewhere (Jiménez-Murcia et al., 2006). Disorder-related variables such as the age of onset and duration of GD were also assessed. Some variables were updated during the lockdown period as part of the *Brief Telephone Survey*, as it was described below.

#### At Lockdown

*Brief Telephone Survey* (Behavioral Addictions Unit, Psychiatry Department, University Hospital, Barcelona, Spain, 2020). A binary qualitative scale (yes/no) was employed to answer most of the points although some questions were open-ended. The different sections of the survey are described as follows:

a. Socio-demographics in the lockdown situation: (1) employment status, (2) working during confinement, (3) being confined and since when with reference to the beginning of the lockdown period, (4) affected close people by COVID-19, and (5) the presence of social support.
b. GD behaviors in the lockdown situation: (1) increased, reduced, or maintained GD symptoms versus abstinence state and (2) the presence of new symptoms related to GD not referred before the lockdown (e.g., different type of gambling modality).
c. COVID-19-related concerns in the lockdown situation: (1) fear of infection, (2) uncertain future, (3) worries about employment, and (4) difficulties in accessing to treatment.
d. Psychological state due to the pandemic (1) pandemic-related anxiety at psychic, emotional, motor, and cognitive dimensions (e.g., headaches, tachycardia, dizziness, nervousness, avoidance behaviors, recurrent thoughts, intrusions, and anticipation) and (2) the presence of depressive-symptoms in the lockdown situation (e.g., presence of hopelessness, pessimism, discouragement, sleepiness, indifference, lack of illusion, crying, social/communicative isolation, and passive thoughts of death).
e. Questions about other items, such as (1) boredom, (2) frequent arguments with cohabitants, family, friends, and (3) receiving and looking for continuous information on COVID-19 were also included.
f. Finally, coping strategies during confinement were evaluated in two ways: (1) as the presence or absence of both adaptive and maladaptive mechanisms (yes/no) and (2) the kind of strategies used, categorizing the coping mechanisms in five dimensions for adaptive strategies (i.e., social contact, leisure, sporting activities, daily routines, and academic/work activity) and three related to maladaptive ones (i.e., obtaining too much COVID-19-related information; behaviors related to GD and other maladaptive behaviors, such as substance use).

### Statistical Analysis

Statistical analysis was conducted with Stata17 for Windows (StateCorp, 2021). First, the incidence density rate (*IDR*, also called the person-time incidence rate) of dropout and relapse was estimated, and comparison between the periods T1 (i.e., during the lockdown) and T2 (i.e., after the lockdown) were performed. *IDR* is the measure of the frequency with which the event occurs (in the study dropout and gambling-episodes) over a specified period. The denominator of this estimation is the product of the person-time of the at-risk sample, and therefore this measurement is not dependent on the duration of the observational period.

Next, chi-square tests (χ^2^) were evaluated comparisons between groups for categorical variables, and T-TEST for quantitative measures. For these analyses, Cohen’s *h* coefficients measured effect sizes for proportion comparisons and Cohen’s *d* for mean comparisons (null effect size was considered for values |*h*|<0.20 or |*d*|<0.20, low-poor for |*h*|>0.20 or |*d*|>0.20, moderate-medium for |*h*|>0.50 or |*d*|>0.50, and large-high for |*h*|>0.80 or |*d*|>0.80; Cohen, 1988; Kelley and Preacher, 2012). In addition, the Finner’s-method (a family-wise error rate stepwise procedure, a less stringent approach than a conservative Bonferroni correction) was used to control Type-I error related to multiple comparisons (Finner and Roters, 2001).

Third, logistic regressions explored significant contributors for the risk of dropout during the observational period, considering as potential predictors socio-demographic variables (sex, age, marital status, education, employment, and social position index), contextual variables during COVID-19-related confinement, GD-related measures at baseline/prior to the confinement (age of onset and duration of gambling problems, and problem-gambling severity), personality measures (TCI-R scores) and psychological distress at baseline (SCL-90R GSI). These analyses used a stepwise method to automatically select significant statistical predictors separately for the risk of dropout during the lockdown (i.e., T1) and after the lockdown (i.e., T2).

## Results

### Characteristics of the Sample

Most participants were men (91.9%), employed (53.5%) and within mean-low to low social position indexes (75.5%). Many were single (44.4%) or married (43.0%), with primary (45.3%) or secondary (40.7%) education levels. The mean age was 45.0years (*SD*=16.9), mean age of onset of GD was 31.8years (*SD*=12.7) and mean duration of GD was 6.2years (*SD*=7.8). The most frequently acknowledged preferred gambling form was non-strategic [46.5%, followed by strategic (33.7%), and mixed (19.8%)], and preferred gambling locations was through offline platforms (66.3%). Table 1 includes the complete description for the variables of the study measured at baseline, prior to the COVID-19-related confinement.

**Table 1:** Socio-demographics and clinical features (*n*=86).

Socio-demographics		*n*	%	Clinical features	Mean	SD
Gender	Male	79	91.9%	Onset of GD (yrs.)	31.77	12.67
	Female	7	8.1%	Duration of GD (yrs.)	6.21	7.78
Marital status	Single	38	44.2%	Duration of treatment and follow-up (months)	14.29	9.19
	Married - in a couple	37	43.0%	^1^SOGS total score	10.73	3.23
	Divorced - separated	11	12.8%	^1^DSM-5 total criteria	7.05	2.11
Education	Primary/less	39	45.3%	SCL-90R: Somatization	0.95	0.82
	Secondary	35	40.7%	SCL-90R: Obsess. /Compulsive	1.21	0.81
	University	12	14.0%	SCL-90R: Interpersonal sensitivity	1.05	0.85
Social position	High / mean-high	12	14.0%	SCL-90R: Depressive	1.53	0.95
	Mean	9	10.5%	SCL-90R: Anxiety	0.98	0.79
	Mean-low	31	36.0%	SCL-90R: Hostility	0.96	0.88
	Low	34	39.5%	SCL-90R: Phobic anxiety	0.45	0.75
Employment	Unemployed	40	46.5%	SCL-90R: Paranoid Ideation	0.91	0.76
	Employed	46	53.5%	SCL-90R: Psychotic	0.98	0.81
Age (yrs.); *mean-SD*		45.00	16.85	SCL-90R: PST score	1.07	0.71
*Gambling-related variables*		*n*	%	SCL-90R: GSI score	46.27	21.59
Gambling type	Non-strategic	40	46.5%	SCL-90R: PSDI score	1.91	0.64
	Strategic	29	33.7%	TCI-R: Novelty seeking	111.72	11.96
	Mixed	17	19.8%	TCI-R: Harm avoidance	98.72	14.01
Modality	Offline	57	66.3%	TCI-R: Reward dependence	97.09	13.50
	Online	12	14.0%	TCI-R: Persistence	107.87	18.69
	Mixed	17	19.8%	TCI-R: Self-directedness	129.92	19.65
Treatment	Individual	27	31.4%	TCI-R: Cooperativeness	128.98	15.36
	Group	59	68.6%	TCI-R: Self-Transcendence	59.94	11.77

*SD: standard deviation. GD: gambling disorder*.

### Environmental Contextual Factors During COVID-19-Related Confinement

Table 2 includes the frequency distribution of the variables assessing contextual environment and personal measures during the COVID-19-related confinement. The percentage of participants reporting working was 25.6%, while 89.5% lived with other people, and 5.8% indicated an affected close people by COVID-19.

**Table 2:** Descriptive variables related to confinement during the COVID-19 pandemic.

	*n*	*%*		*n*	*%*
Working	22	25.6%	Non-adaptive reactions	19	22.1%
With company	77	89.5%	Non-adaptive reactions: COVID	11	12.8%
Affected close people by COVID	5	5.8%	Non-adaptive reactions: GD	1	1.2%
COVID-related concerns	58	67.4%	Non-adaptive reactions: Other	7	8.1%
Concerns: infection risk	35	40.7%	Anxiety (any type)	22	25.6%
Concerns: uncertain future	34	39.5%	Anxiety: physic	10	11.6%
Concerns: employment	19	22.1%	Anxiety: emotional	17	19.8%
Concerns: medical treatments	2	2.3%	Anxiety: motor	6	7.0%
Concerns: other	5	5.8%	Anxiety: cognitive	16	18.6%
Adaptive reactions	64	74.4%	Depression	17	19.8%
Adaptive reactions: social	21	24.4%	Family conflicts	8	9.3%
Adaptive reactions: leisure	30	34.9%			
Adaptive reactions: sport	10	11.6%			
Adaptive reactions: routine	25	29.1%			
Adaptive reactions: work-studies	5	5.8%			

*SD: standard deviation*.

The percentage of participants reporting COVID-19-related concerns was 67.4%. Most patients employed adaptive reactions (74.4%), while 22.1% reported maladaptive reactions. The presence of anxiety symptoms was reported by 25.6% of the participants, and depressive symptoms by 19.8%. Family conflicts due to confinement were reported by 9.3% of participants.

### Factors Contributing to Dropout During the Observational Period

**Figure 1:**
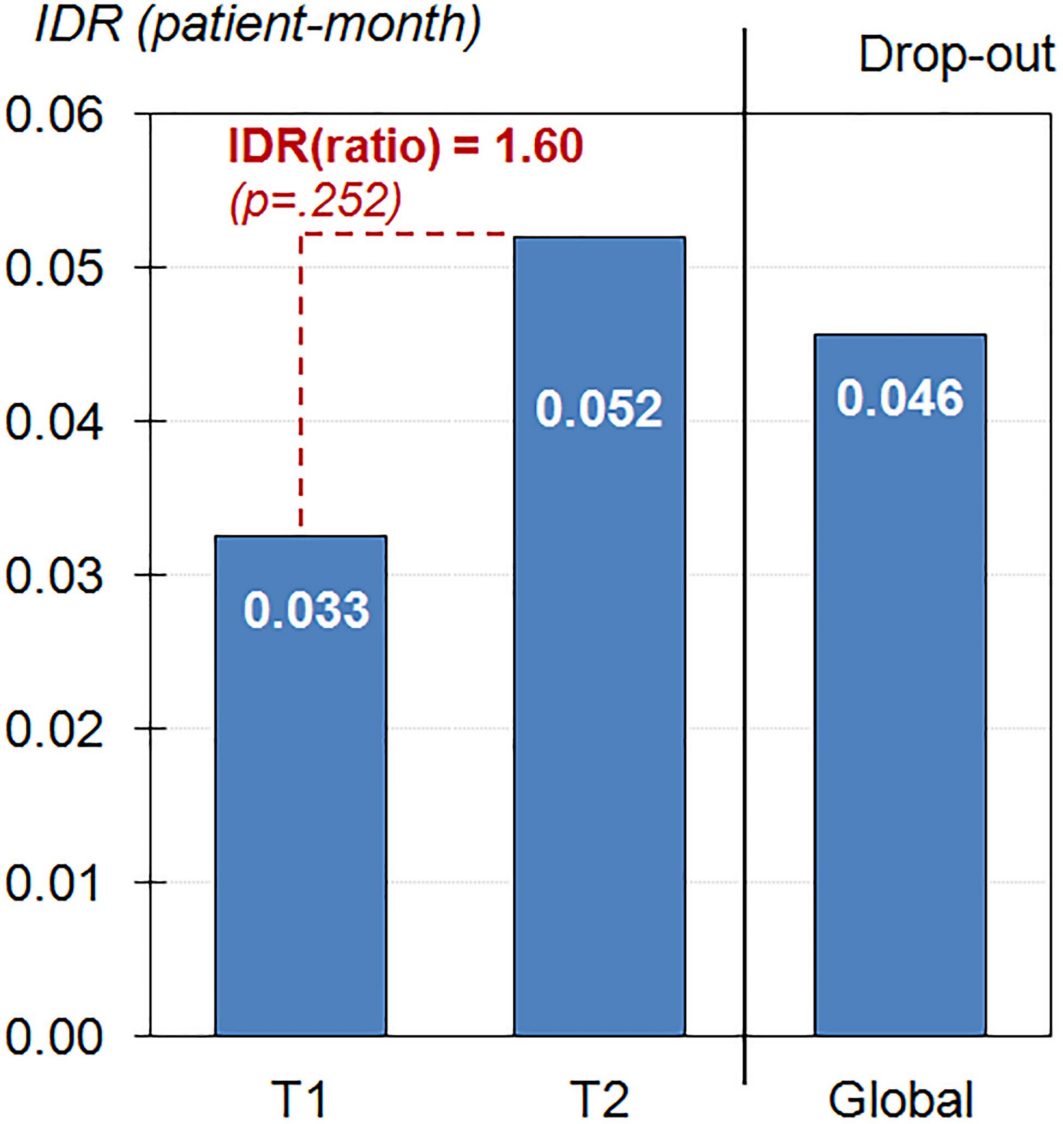
Incidence Density Rate (IDR) of Dropout during the nine-month observational period (*n*=86).

The risk of dropout during the complete observational period was *R*=32/86=0.372 (37.2%), and the *IDR* was 0.045 per person month (4.5 patients dropped out per 100 participants-month). Considering separately the two periods of time of the observational period (Figure 1), *IDR* increased from 0.033 (3.3 individuals dropped out per 100 participants-month) at T1 to 0.052 (5.2 dropped out per 100 participants-month) at T2, the *IDR* ratio being equal to 0.052/0.033=1.60 (*p*=0.252).

**Figure 2:**
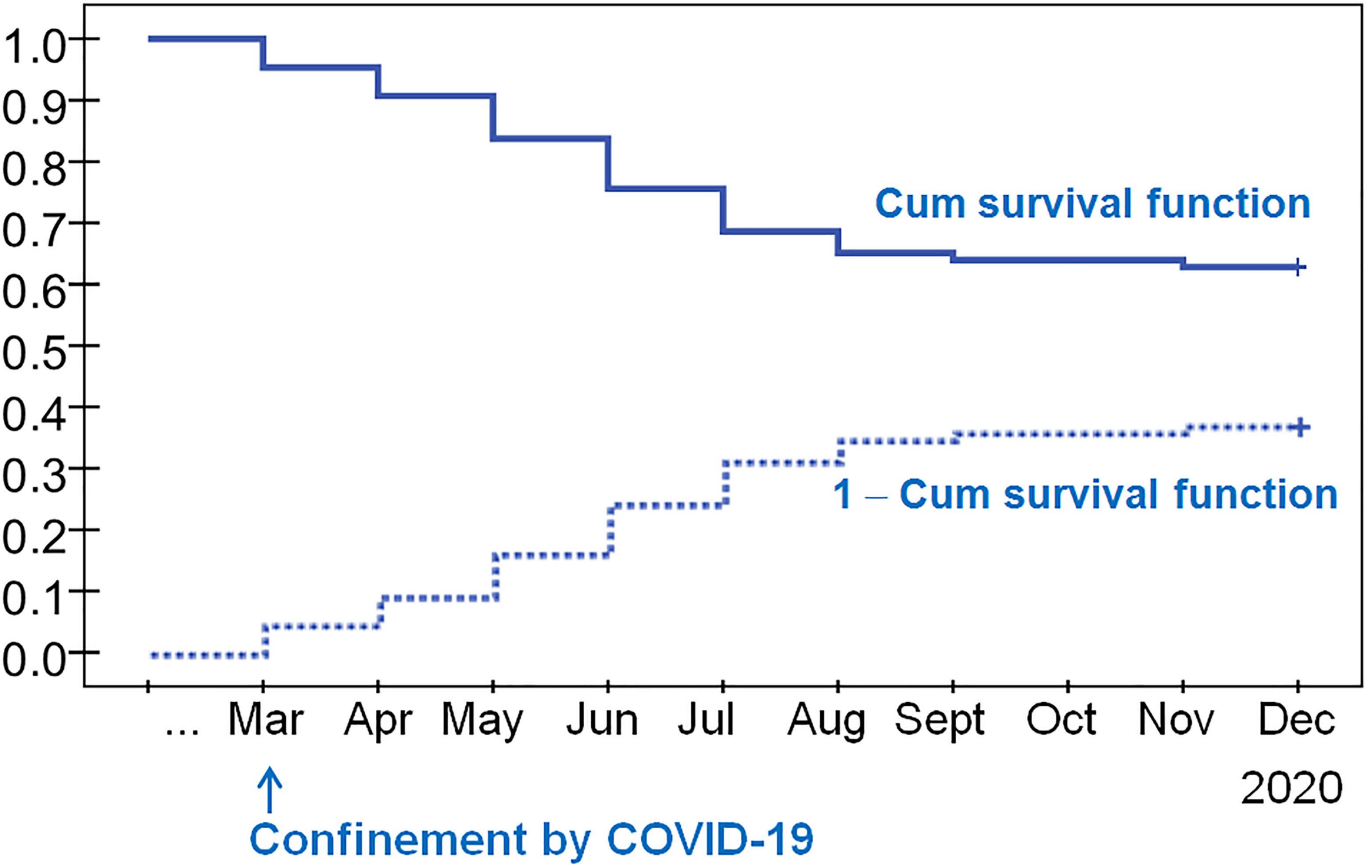
Kaplan–Meier survival function to dropout (*n*=86).

Figure 2 shows the survival functions (Kaplan–Meier product-limit estimator) for dropouts, considering the period from the regional COVID-19-related the lockdown and the end of the observational period (March 2020–December 2020). The curve in the upper part of the figure (continuous-line) represents the cumulative survival function, and the lower curve (dash-line) the inverse 1-cumulative survival function. The cumulate survival function estimates the proportion of patients *surviving* in treatment (without dropout) for certain amounts of time. The inverse curve (1-cumulative survival function) plots the cumulate proportion of dropouts during the observational period. The shape of the cumulate survival function, in the form of regular jumps/rectangles during the period March–July suggested that most dropouts were observed during this period, (concretely, 31.4% of participants had abandoned during these months). The remaining 5.8% of dropouts were registered during the following months (August–December).

**Figure 3:**
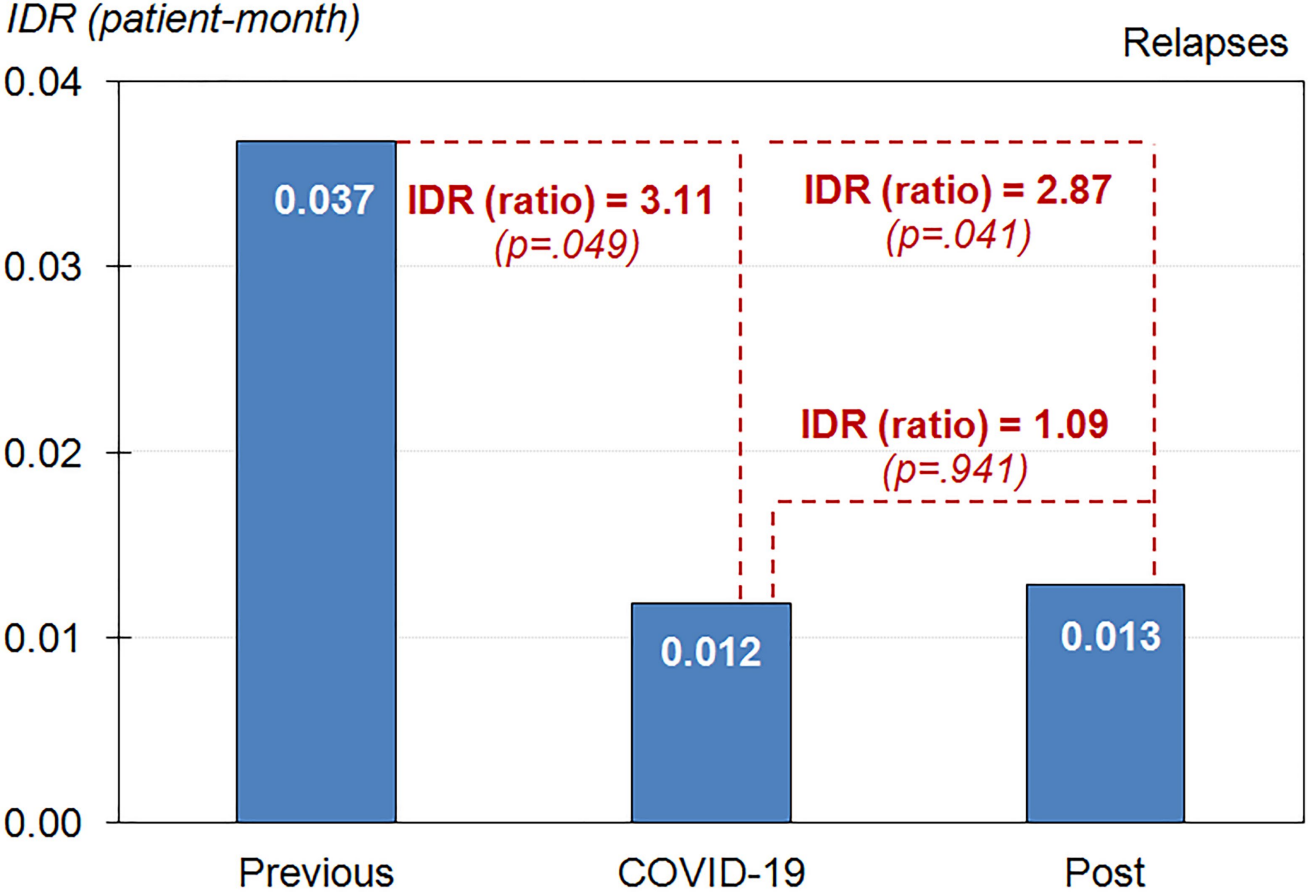
Incidence Density Rate (IDR) of relapse rates during the nine-month observational period (*n*=86).

The frequency of relapses was very low during the complete observational period. Figure 3 contains the bar-chart with the *IDR* of gambling episodes in the study. The lowest *IDR* was registered during the lockdown period (1.2 patients reported relapses per 100 participants-month), followed by the post-lockdown stage (1.3 patients reported relapses per 100 participants-month). Compared with the period prior to when confinement started (*IDR*=0.037 at baseline, 3.7 relapses per 100 participants-month), the probability of relapses during the lockdown was 3.11 times lower (*p*=0.049), and after the lockdown the probability of relapses was 2.87 times lower (*p*=0.041). No statistical differences was observed comparing the *IDR* during and after the lockdown (*p*=0.941).

Table 3 shows the bivariate comparisons between the groups defined based on the dropouts (non-dropout versus dropout) for the variables of the study (socio-demographics, gambling related measures, clinical profile, and those variables collected *via* the *Brief Telephone Survey*). Separate analyses were performed for the T1 and T2 stages. Variables related to the increase in the risk of dropout during T1 were male sex, higher education levels, higher social position indexes, lower SCL-90R anxiety and psychotic scores, lower use of adaptive and non-adaptive reactions, and lower level of anxiety and depression symptoms due to pandemic. Online gambling was a protective factor for dropout during T1. Variables contributing to increase the risk of dropout during T2 were shorter duration of treatment and more concerns related to employment status during the lockdown.

**Table 3:** Association between socio-demographics and dropout during two periods of the COVID-19 pandemic: during (T1) and after (T2) the lockdown.

		T1						T2					
		Non-dropout *n*=78		Dropout *n*=8				Non-dropout *n*=54		Dropout *n*=24			
*Socio-demographics*		*n*	*%*	*n*	*%*	*p*	*|h|*	*n*	*%*	*n*	*%*	*p*	*|h|*
Gender (female)		7	9.0%	0	0.0%	0.377	**0.61** ^**†**^	5	9.3%	2	8.3%	0.895	0.03
Marital status (non-married)		46	59.0%	3	37.5%	0.243	0.43	30	55.6%	16	66.7%	0.357	0.23
Education (low levels)		37	47.4%	2	25.0%	0.225	**0.51** ^**†**^	26	48.1%	11	45.8%	0.850	0.05
Social status (low levels)		33	42.3%	1	12.5%	0.043_*_	**0.69** ^**†**^	22	40.7%	11	45.8%	0.674	0.10
Employment (unemployed)		38	48.7%	2	25.0%	0.200	0.48	26	48.1%	12	50.0%	0.880	0.04
*Gambling activity*		*n*	*%*	*n*	*%*	*p*	*|h|*	*n*	*%*	*n*	*%*	*p*	*|h|*
Type	Non-strategic	36	46.2%	4	50.0%	0.842	0.08	26	48.1%	10	41.7%	0.328	0.13
	Strategic	27	34.6%	2	25.0%		0.21	16	29.6%	11	45.8%		0.34
	Mixed	15	19.2%	2	25.0%		0.14	12	22.2%	3	12.5%		0.26
Modality	Offline	52	66.7%	5	62.5%	0.265	0.09	37	68.5%	15	62.5%	0.538	0.13
	Online	12	15.4%	0	0.0%		**0.81** ^**†**^	9	16.7%	3	12.5%		0.12
	Mixed	14	17.9%	3	37.5%		0.44	8	14.8%	6	25.0%		0.26
*Clinical profile*		*Mean*	*SD*	*Mean*	*SD*	*p*	*|d|*	*Mean*	*SD*	*Mean*	*SD*	*p*	*|d|*
Age (yrs)		44.74	16.67	47.50	19.61	0.662	0.15	45.74	16.59	42.50	16.97	0.432	0.19
Onset of GD (yrs)		31.96	12.90	29.94	10.70	0.669	0.17	32.81	12.98	30.06	12.76	0.387	0.21
Duration of GD (yrs)		6.47	8.04	3.63	3.89	0.327	0.45	6.80	8.22	5.75	7.76	0.599	0.13
Duration of treatment (months)		15.14	9.17	6.00	3.59	**0.007**	**1.31** ^**†**^	18.09	9.16	8.50	4.66	**0.001**	**1.32** ^**†**^
^1^SOGS total score		10.78	3.29	10.25	2.66	0.660	0.18	10.63	3.29	11.13	3.34	0.543	0.15
^1^DSM-5 total criteria		7.04	2.18	7.13	1.25	0.913	0.05	7.17	2.25	6.75	2.03	0.440	0.19
SCL-90R: Somatization		0.96	0.83	0.83	0.69	0.675	0.17	0.91	0.81	1.07	0.88	0.434	0.19
SCL-90R: Obsess. /comp.		1.24	0.79	0.90	0.97	0.263	0.38	1.25	0.76	1.20	0.87	0.808	0.06
SCL-90R: Interp.sens.		1.08	0.85	0.74	0.84	0.274	0.41	1.06	0.79	1.14	0.97	0.691	0.09
SCL-90R: Depressive		1.56	0.94	1.17	1.06	0.271	0.39	1.58	0.90	1.53	1.05	0.812	0.06
SCL-90R: Anxiety		1.02	0.80	0.61	0.58	0.167	**0.58** ^**†**^	1.06	0.76	0.92	0.89	0.473	0.17
SCL-90R: Hostility		0.99	0.88	0.73	0.87	0.433	0.29	0.97	0.80	1.03	1.07	0.753	0.07
SCL-90R: Phobic anx.		0.47	0.78	0.21	0.24	0.358	0.45	0.51	0.83	0.39	0.65	0.529	0.16
SCL-90R: Paranoid		0.93	0.77	0.69	0.66	0.393	0.34	0.89	0.71	1.03	0.92	0.469	0.17
SCL-90R: Psychotic		1.02	0.82	0.59	0.59	0.152	**0.60** ^**†**^	0.98	0.73	1.10	1.00	0.578	0.13
SCL-90R: PST score		1.10	0.72	0.79	0.67	0.242	0.45	1.10	0.66	1.11	0.84	0.954	0.01
SCL-90R: GSI score		47.27	21.06	36.50	25.67	0.181	0.46	47.50	19.16	46.75	25.27	0.886	0.03
SCL-90R: PSDI score		1.94	0.65	1.69	0.57	0.304	0.40	1.97	0.62	1.86	0.72	0.492	0.16
TCI-R: Novelty seeking		111.82	12.40	110.75	6.71	0.811	0.11	111.33	12.55	112.92	12.25	0.606	0.13
TCI-R: Harm avoidance		98.77	13.65	98.25	18.29	0.921	0.03	99.39	13.23	97.38	14.74	0.551	0.14
TCI-R: Reward depend.		96.92	13.32	98.75	16.02	0.718	0.12	98.30	13.57	93.83	12.46	0.174	0.34
TCI-R: Persistence		107.82	18.11	108.38	25.21	0.937	0.03	108.17	19.54	107.04	14.72	0.802	0.07
TCI-R: Self-directedness		129.15	19.79	137.38	17.68	0.262	0.44	128.89	18.08	129.75	23.60	0.861	0.04
TCI-R: Cooperativeness		128.53	15.66	133.38	12.07	0.398	0.35	129.54	15.48	126.25	16.15	0.396	0.21
TCI-R: Self-Transcend.		59.91	12.24	60.25	6.02	0.939	0.04	59.89	11.33	59.96	14.34	0.982	0.01
*Confinement measures*		*n*	*%*	*n*	*%*	*p*	*|h|*	*n*	*%*	*n*	*%*	*p*	*|h|*
Working		20	25.6%	2	25.0%	0.968	0.01	14	25.9%	6	25.0%	0.931	0.02
With company		71	91.0%	6	75.0%	0.158	0.44	48	88.9%	23	95.8%	0.322	0.27
Affected family environment		4	5.1%	1	12.5%	0.396	0.27	2	3.7%	2	8.3%	0.392	0.20
COVID-related concerns		53	67.9%	5	62.5%	0.754	0.11	37	68.5%	16	66.7%	0.872	0.04
Concerns: infection risk		31	39.7%	4	50.0%	0.574	0.21	21	38.9%	10	41.7%	0.909	0.06
Concerns: uncertain future		32	41.0%	2	25.0%	0.377	0.34	23	42.6%	9	37.5%	0.673	0.10
Concerns: employment		18	23.1%	1	12.5%	0.492	0.28	9	16.7%	9	37.5%	**0.044**	**0.51** ^**†**^
Adaptive reactions		60	76.9%	4	50.0%	**0.046**	**0.57** ^**†**^	42	77.8%	18	75.0%	0.788	0.07
Adaptive reactions: social		19	24.4%	2	25.0%	0.968	0.01	14	25.9%	5	20.8%	0.629	0.12
Adaptive reactions: leisure		27	34.6%	3	37.5%	0.870	0.06	20	37.0%	7	29.2%	0.500	0.17
Adaptive reactions: routines		24	30.8%	1	12.5%	0.278	**0.51** ^**†**^	15	27.8%	9	37.5%	0.391	0.21
Non-adaptive reactions		19	24.4%	0	0.0%	**0.040**	**1.03** ^**†**^	14	25.9%	5	20.8%	0.629	0.12
Anxiety		22	28.2%	0	0.0%	**0.025**	**1.12** ^**†**^	15	27.8%	7	29.2%	0.900	0.03
Depression		17	21.8%	0	0.0%	**0.045**	**0.97** ^**†**^	14	25.9%	3	12.5%	0.185	0.35

*SD: standard deviation*.

† ***Bold**: effect size into the range mild–moderate (|d|>0.50 or |h|>0.50) to high-large (|d|>0.80 or |h|>0.80)*.

Table 4 contains the final models obtained in the stepwise logistic regression models, considering the risk of dropout as criterion and as potential predictors the socio-demographic variables, the gambling related measures at baseline, clinical state at baseline and during the lockdown, as well as the contextual factors during the lockdown. The three final models obtained considering separately the stages T1 and T2, and the entire observational period, retained as the only significant predictor the duration of the GD treatment: the shorter the duration of treatment, the higher the likelihood of dropout.

**Table 4:** Predictive models for the risk of dropout: stepwise logistic regression (*n*=86).

	*B*	*SE*	*p*	*OR*	*95%CI (OR)*	
Dropout during T1 Duration of treatment (months)	−0.341	0.120	0.005	0.711	0.561	0.900
Dropout during T2						
Duration of treatment (months)	−0.349	0.090	<0.001	0.706	0.592	0.841
Dropout (during T1 or T2)						
Duration of treatment (months)	−0.382	0.090	<0.001	0.682	0.572	0.814

*List of predictors: socio-demographics, variables at baseline and variables related to the confinement by COVID-19*.

## Discussion

The present study was designed to explore the impact of the COVID-19 pandemic and confinement on treatment adherence among patients with GD. For this purpose, we evaluated dropout rates both during and after the lockdown. The relationship between clinical variables and dropout was also explored in both periods.

Most patients of the sample were male and employed, and their main gambling problem involved offline gambling. This socio-demographic and clinical profile is similar to the characterization reported in previous studies of our Unit (Jiménez-Murcia et al., 2007, 2015). Regarding treatment outcome, the risk of dropout over the nine-month observational period reached similar values to those mentioned in the literature (Melville et al., 2007) and described before confinement started (Jiménez-Murcia et al., 2019). A slightly lower mean incidence of dropout was found *during the lockdown* in comparison with the following months although without reaching statistical significance. The restrictive characteristics of the lockdown has allowed for greater stimulus control, especially related to offline gambling activity. Consequently, the reduction of gambling opportunities, improved social/familiar environments (Donati et al., 2021), and the maintenance of an active treatment adapted to the context might be considered as protective factors (e.g., reassessing the situation and seeking alternative methods of treatment and prevention; Lee and Rovers, 2008; Côté et al., 2020).

Lower levels of anxiety and depressive symptoms due to pandemic and higher socio-economic positions at baseline were stated among the individuals who dropped out *during the lockdown*. These findings possibly suggest that those patients with better socio-economic background prior to confinement and lower referred psychological impact due to pandemic (in terms of anxiety and depressive symptoms) may have been more likely to discontinue treatment, already from the initial stages of confinement. Also, a lower use of adaptive coping strategies during the lockdown was also described in patients who dropped out in comparison to those who remained in treatment. In contrast, patients with greater insight about their gambling problem and more urgent needs for professional help may have been more likely to remain in treatment, even if the need was not directly related to gambling behavior (i.e., social support, anxiety, and depressive symptoms). Several individuals who remained in treatment reported using non-adaptive strategies during the lockdown (such as concerns related to COVID-19 and gambling behavior). In this sense, we hypothesize that treatment attendance may represent an adaptive mechanism to deal with emotional distress. Hence, treatment should focus on coping with emotions as a helpful approach to tackle gambling problems in the face of similar future adverse circumstances (Estévez et al., 2021).

*During the lockdown*, a more flexible approach to treatment was employed (Marsden et al., 2020), using Internet-based tools therapeutic strategies (Columb et al., 2020). This approach may have influenced treatment adherence in patients who were already in treatment for GD, from two different perspectives. On the one hand, online therapy was more accessible to people who had previously found it difficult to attend (American Psychological Association, 2015). Moreover, the relationship between therapist and patient in the case of virtual therapy has been described as good as for in-person therapy (Bergman and Kelly, 2021), allowing the therapeutic alliance to be preserved in these exceptional circumstances. On the other hand, the absence of in-person activities, a lack of peer-to-peer social and emotional connections, inequities in Internet access, and online distractions could represent challenges for adapting to shifting from face-to-face to online therapy. These issues have been previously reported in virtual therapeutic approaches in other mental disorders during confinement, such as in substance use disorders and eating disorders (Fernández-Aranda et al., 2020b; Bergman and Kelly, 2021). In the present study, although changes in the treatment approach did not seem to have detrimental effects on treatment adherence, some of the patients who dropped out may have presented some of the described difficulties. Pandemic situation highlights the need to efficiently adapt the available treatment strategies to these exceptional circumstances to ensure therapeutic continuity. In this vein, customizing proven treatment strategies to virtual formats has been a useful tool, preserving the initial therapeutic objectives.

*After the lockdown*, restrictive measures were progressively reduced (e.g., related to “stay-at-home” measures, social distancing, and diminished mobility) and the treatment program in its original format was reinstated. Dropout was associated with more employment concerns due to the pandemic. In this line, the financial pressure of job insecurity and unemployment could motivate individuals to use gambling as a mechanism to earn money and manage debts (van Schalkwyk et al., 2020; Salerno and Pallanti, 2021). Also, previous financial crises have shown the possibility of engaging in gambling behaviors to attempt economic restitution (Olason et al., 2015; Economou et al., 2019). One plausible explanation for our results could be that those patients reporting work instability and difficult financial situations due to the lockdown could have relapsed and abandoned treatment. Another possible rationale could be that, coinciding with the easing of restrictions after the lockdown, some patients may find it difficult to juggle work and treatment, prioritizing the search and maintenance of a job over treatment. While many of these notions are currently speculative, they warrant direct examination in future studies.

The survival analysis showed that most dropouts were concentrated in the first months of this period, especially between May and July 2020. These months in Spain could be considered as a transition period between the measures taken during the lockdown and the beginning of the gradual phasing out of these measures. It was characterized by an increased uncertainty about the return to daily routines (e.g., work routines), the ubiquity of social contact and health concerns related to COVID-19. The general instability of this phase, along with the reopening of the gambling establishments, made these first months after lockdown a particular period of high vulnerability for relapse and for treatment dropout. Furthermore, some of the patients who abandoned treatment during the first months of confinement were in the early stages of the treatment phase. These results are in line with previous studies which described a predominant tendency to dropout at the start of the treatment or after the first few sessions (Jiménez-Murcia et al., 2015).

Going one step further, mean incidence of relapse for gambling behavior was significantly decreased during and after lockdown in comparison with mean rates prior to the confinement started. However, there were not differences in relapse rates between during and after the lockdown. Most patients reported mainly gambling offline and living with others during the nine-month observational period. A high abstinence from gambling of around 75% has previously been described in patients who were already in CBT treatment and/or in the follow-up phase (Jiménez-Murcia et al., 2007; Cowlishaw et al., 2012). In the context of pandemic, our results also agreed with recent literature that pointed out higher abstinence rate during confinement (Avanzi et al., 2020). In line with Donati et al. (2021), restrictive measures due to pandemic, social support and some policies related to gambling activity adopted during confinement, have promoted gambling abstinence by reducing offline gambling opportunities.

Finally, an association between treatment duration and dropout was found during and after lockdown. Specifically, the shorter the duration of treatment, the higher the risk of dropout during and after the lockdown. Furthermore, and according to the predictive analysis model, the duration of the treatment would be a predictive factor of dropout. Length of therapy has been positively related to treatment outcome in previous studies (Orlinsky et al., 1994). In this sense, achieving a strong therapeutic alliance with the patient early in treatment is important for improving adherence to therapy (Gómez-Peña et al., 2012). Therefore, patients with weaker therapeutic links and lower durations of treatment may have found it more challenging to preserve treatment adherence in the face of similar adverse situations.

## Strengths and Limitations

The strengths of the study included a clinical sample of patients already engaged with our treatment Unit. Moreover, we used clinical records and assessments to evaluate patients rather than just self-report measures. The longitudinal nature of this work allowed reporting treatment outcomes related to data over the course of 9months. Limitations included the modest sample size, limited geographic location, and the absence of a control group for comparison and multiple qualitative assessments. Some clinical and socio-demographic variables were collected prior to the onset of the lockdown (i.e., at the baseline evaluation prior to treatment) and their possible changes due to the pandemic were updated in the short-term during confinement. However, the influence of new changes in these variables in the middle-long term should be contemplated in future research. Moreover, upcoming studies would benefit from including quantitative validated scales and evaluating changes over longer time periods in GD symptomatology and associated factors, throughout different stages of the COVID-19 pandemic in multiple jurisdictions and cultures.

## Conclusion

In conclusion, the present findings provide data on the treatment dropout in clinical population diagnosed with GD and potential contributing factors during confinement. In this line, duration of treatment could be a possible predictor of dropout in the face of adverse external situations. These results have relevance for identifying potentially high-risk subjects and optimizing individualized early interventions in the setting of future similar adverse circumstances.

## Data Availability Statement

Individuals may inquire with Dr. Jiménez-Murcia regarding availability of the data as there is ongoing studies using the data. To avoid overlapping research efforts, Dr. Jiménez-Murcia will consider a request on a case-by-case basis.

## Ethics Statement

The present study was conducted in accordance with the latest version of the Declaration of Helsinki. The Clinical Research Ethics Committee of the first author’s Public University Hospital gave approval before confinement, as most of the data used in this study was part of the baseline 308 assessment protocol prior to the start of treatment and previously used in the Unit’s studies related to treatment (Ref: PR329/19). We added the brief telephone survey to this protocol at the beginning of confinement. Signed informed consent was obtained from all participants.

## Author Contributions

IB, EC, ME, and SJ-M contributed to the development of the study concept and design and aided with interpretation of data and the writing of the manuscript. RG performed the statistical analysis. IB, MG-P, LM, SR, AP-G, BM-M, EV-M, and SJ-M aided with data collection. FF-A, SJ-M, MP, AH, and JM revised the manuscript and provided substantial comments. FF-A and SJ-M obtained funding. All authors contributed to the article and approved the submitted version.

## Funding

CERCA Programme/Generalitat de Catalunya gave institutional support. This work was additionally supported by a grant from the Ministerio de Ciencia, Innovación y Universidades (grant RTI2018-101837-B-100). The research was funded by the Delegación del Gobierno para el Plan Nacional sobre Drogas (2017I067 and 2019I47), Instituto de Salud Carlos III (ISCIII) (PI17/01167) and co-funded by FEDER funds/European Regional Development Fund (ERDF), a way to build Europe. CIBEROBN and CIBERSAM are both initiatives of ISCIII. IB was partially supported by a Post-Residency Grant from Research Committee of the University Hospital of Bellvitge (HUB; Barcelona, Spain) 2020–2021. The funders had no role in the study design, data collection and analysis, decision to publish or preparation of the manuscript.

## Conflict of Interest

AH holds a position at Lund University, Sweden, sponsored by the state-owned Swedish gambling operator AB Svenska Spel and has funding from the research council of the same organization and from the research councils of the Swedish state-owned alcohol monopoly Systembolaget AB and the Swedish sports federation. MP notes the following disclosures. He has: consulted for and advised Game Day Data, the Addiction Policy Forum, AXA, Idorsia, and Opiant/Lakelight Therapeutics; received research support from the Veteran’s Administration, Mohegan Sun Casino, and the National Center for Responsible Gaming (on the International Center for Responsible Gambling); participated in surveys, mailings, or telephone consultations related to addictions, impulse-control disorders or other health topics; consulted for law offices and the federal public defender’s office in issues related to impulse-control and addictive disorders; provided clinical care in the Connecticut Department of Mental Health and Addiction Services Problem Gambling Services Program; performed grant reviews for the National Institutes of Health and other agencies; edited journals and journal sections; given academic lectures in grand rounds, CME events and other clinical/scientific venues; and generated books or chapters for publishers of mental health texts.

The remaining authors declare that the research was conducted in the absence of any commercial or financial relationships that could be construed as a potential conflict of interest.

## Publisher’s Note

All claims expressed in this article are solely those of the authors and do not necessarily represent those of their affiliated organizations, or those of the publisher, the editors and the reviewers. Any product that may be evaluated in this article, or claim that may be made by its manufacturer, is not guaranteed or endorsed by the publisher.
